# Alterations in the fecal microbiota of methamphetamine users with bad sleep quality during abstinence

**DOI:** 10.1186/s12888-024-05773-5

**Published:** 2024-04-25

**Authors:** Zijing Deng, Linzi Liu, Wen Liu, Ruina Liu, Tao Ma, Yide Xin, Yu Xie, Yifan Zhang, Yifang Zhou, Yanqing Tang

**Affiliations:** 1 Brain Function Research Section, The First Hospital of China Medical University, Shenyang, 110001 Liaoning PR China; 2 Department of Psychiatry, The First Hospital of China Medical University, Shenyang, 110001 Liaoning PR China; 3 Department of Psychiatry, Shengjing Hospital of China Medical University, Shenyang, 110004 Liaoning PR China; 4 Department of Psychiatry, The First Affiliated Hospital of Xi’an Jiaotong University, Xi’an, 710061 Shanxi PR China

**Keywords:** Methamphetamine, Sleep quality, Gut microbiota, Microbiota-gut-brain axis

## Abstract

**Background:**

Methamphetamine (MA) abuse has resulted in a plethora of social issues. Sleep disturbance is a prominent issue about MA addiction, which serve as a risk factor for relapse, and the gut microbiota could play an important role in the pathophysiological mechanisms of sleep disturbances. Therefore, improving sleep quality can be beneficial for treating methamphetamine addiction, and interventions addressing the gut microbiota may represent a promising approach.

**Method:**

We recruited 70 MA users to investigate the associations between sleep quality and fecal microbiota by the Pittsburgh Sleep Quality Index (PSQI), which was divided into MA-GS (PSQI score < 7, MA users with good sleep quality, *n* = 49) and MA-BS group (PSQI score ≥ 7, MA users with bad sleep quality, *n* = 21). In addition, we compared the gut microbiota between the MA-GS and healthy control (HC, *n* = 38) groups. 16S rRNA sequencing was applied to identify the gut bacteria.

**Result:**

The study revealed that the relative abundances of the *Thermoanaerobacterales* at the order level differed between the MA-GS and MA-BS groups. Additionally, a positive correlation was found between the relative abundance of the genus *Sutterella* and daytime dysfunction. Furthermore, comparisons between MA users and HCs revealed differences in beta diversity and relative abundances of various bacterial taxa.

**Conclusion:**

In conclusion, the study investigated alterations in the gut microbiota among MA users. Furthermore, we demonstrated that the genus *Sutterella* changes may be associated with daytime dysfunction, suggesting that the genus *Sutterella* may be a biomarker for bad sleep quality in MA users.

**Supplementary Information:**

The online version contains supplementary material available at 10.1186/s12888-024-05773-5.

## Introduction

Methamphetamine (MA), commonly known as ice drug, is a highly prevalent drug worldwide and has caused substantial social problems. In China, at the end of 2019, MA had become the most frequently abused drug, with 1.18 million MA users, accounting for 55.5% of all drug users nationwide [1]. Research has shown that MA use can have negative effects on physical health, including increased risks of tachycardia, hypertension, and rhabdomyolysis [2]. Additionally, MA use may result in mortality due to pulmonary edema, pulmonary congestion, ventricular fibrillation, acute cardiac failure, or hyperpyrexia [2]. Furthermore, MA addiction, compared to MA use without addiction, can lead to altered mental states such as severe depression [3, 4], anxiety [5], and psychiatric symptoms [6, 7] compared to non-MA dependent users. High impulsivity [8], aggression [9], or violent behaviors [10] are also common among individuals addicted to MA.

Sleep disturbance, an important issue related to MA addiction, has garnered considerable attention. Tang et al. found that drug users were more likely to experience sleep disturbances than non-users, with a prevalence rate of 54.16% among MA users [11]. Moreover, sex differences in sleep problems among MA abusers cannot be overlooked, with prevalence rates of MA-related sleep disturbance of 52.4% for males and 75.6% for females [12]. Mahoney et al. found that daytime sleepiness increased in participants with methamphetamine use disorder (MUD) and that they had significantly higher Pittsburgh Sleep Quality Index (PSQI) scores [13]. Another study from Perez et al. revealed that a single intranasal MA dose reduced subjective sleep quality [14]. The literature holds that acute and long-term sleep disturbance may be a cause of addiction relapse [15] and are universal risk factors for psychoactive substance relapse [16]. Sleep disturbances are strongly correlated with violent and aggressive behavior [17, 18]. Wang et al. discovered that melatonin effectively treats sleep disorders caused by MA and can reverse aggression induced by the drug [19]. Therefore, treating sleep disorders in MA users is critical for addiction recovery.

Currently, the roles of the gut microbiota and the microbiota-gut-brain axis in different diseases have attracted extensive attention, especially in neuropsychiatric disorders. Schizophrenia [20, 21], bipolar disorder [22, 23], depression [24], autism spectrum disorder [25], and substance use disorder [26] are associated with the gut microbiota. The microbiota-gut-brain signaling pathway has several mechanisms according to recent studies [27]. The vagal pathway is an important avenue regarding alterations in the gut-brain axis and downstream behaviors [26, 28]. Similarly, the immune system was seen as a central mediator of gut-brain communication [29]. Another component of the microbiota-gut-brain axis is metabolites from the gut microbiota, which consist of short-chain fatty acids [30], bile acids [31], and neurotransmitters [32]. Nevertheless, the potential mechanisms by which the gut microbiota influence brain function have not been fully elucidated.

Alterations in the intestinal microbiota in MA abusers have been confirmed. Yang et al. found decreasing relative abundances in *Deltaproteobacteria* and *Bacteroidaceae* and increasing relative abundances in *Sphingomonadales, Xanthomonadales, Romboutsia*, and *Lachnospiraceae* for the MA users, and cognitive assessment was positively related to *Blautia* [33]. Deng et al. revealed that relative abundances of *Collinsella*, *Odoribacter*, and *Megasphaera* are growing and the levels of *Faecalibacterium*, *Blautia*, *Dorea* and *Streptococcus* were reduced at the genus level in subjects with MA addiction [34]. In addition to the alteration of the gut microbiota, Yang et al. also demonstrated oral microbiota (*Negativicutes*, *Veillonellaceae*, *Veillonella*, and *Selenomonadales*) had higher relative abundance in the MA group which are connected with oral diseases [35]. Another study suggested that the interrelationships between the oral and gut microbiomes sustained attention [36]. In addition, animal experiments have also found MA-induced alterations in the gut microbiota. Chen et al. discovered that the relative abundances of pathogenic bacteria improved while those of probiotics were reduced by MA exposure, with corresponding metabolomics alterations [37]. Wang et al. revealed that MA-induced mice treated with antibiotics exhibit weaker conditioned place preference (CPP), but CPP formation was erased by fecal microbiota transplantation [38]. The above studies suggest that the psychological and behavioral changes induced by MA may be related to gut microbiota.

Regarding the pathophysiological mechanisms of sleep disturbance, the role of the gut microbiota has gradually attracted increasing attention. Zhang et al. found that *Tenericutes*, *Elusimicrobia*, butanoate metabolism, and propanoate metabolism were the main differences between groups with poor and normal sleep quality [39]. Beyond the difference between healthy controls and insomnia patients, significant differences in taxa such as *Lachnospira, Faecalibacterium*, and *Blautia* were observed between chronic and acute insomnia patients [40]. In young healthy individuals, self-reported sleep quality was associated with microbial diversity [41] while in preschool-aged children, a novel association between sleep and gut microbiota was revealed [42]. These experiments all verified abnormalities in the gut microbiota are evident in sleep disorders.

However, previous research on the relationship between the gut microbiota and sleep quality in MA users is lacking. Hence, our goal was to investigate the alterations in the gut microbiota in MA users with bad sleep quality by employing 16S rRNA sequencing. Based on the above results, we speculated that the gut microbiota in MA users with bad sleep quality would exhibit unique characteristics. In addition, we also explored the differences between MA users with good sleep quality and healthy controls.

## Materials and methods

### Study design

In the present research, 80 MA users and 50 healthy controls (HCs) whose ages ranged from 18 to 65 years were recruited from October 2021 to December 2022. The MA users were recruited from the First Compulsory Drug Rehabilitation Center of Shenyang, while HCs were recruited through advertising from the local community. According to the following criteria, 22 participants were excluded due to use of antibiotics, probiotics, or defecation drugs; diabetes; cirrhosis of the liver; or refusing to provided provide stool samples. Finally, a total of 70 MA users and 38 HCs were included in the current study. 70 MA users were divided into two groups, namely the group with good sleep quality and the group with poor sleep quality. The differences of gut microbiota between the two groups were compared and further found the gut microbiota related to sleep quality in MA users. More details of the research will be mentioned later. The Medical Research Ethics Committee of the First Affiliated Hospital of China Medical University approved the study (No. [2021]361). All the participants in the study voluntarily provided written informed consent.

### Inclusion criteria

For MA users, the inclusion criteria involved meeting the Diagnostic and Statistical Manual of Mental Disorders, fifth edition (DSM-5), criteria for MUD and at least two positive urine tests more than one month apart. For HCs, participants with a Pittsburgh Sleep Quality Index (PSQI) score < 7 were included.

### Exclusion criteria

The following exclusion criteria were applied to all participants: (1) other psychiatric diseases such as depressive disorder, bipolar disorder, or schizophrenia (for MA users) and any Axis I or Axis II disorders (for HCs); (2) use of other illicit drugs such as heroin; (3) metabolic disease, autoimmune disease, diabetes, hepatitis, cirrhosis of the liver, or infection with human immunodeficiency virus (HIV); (4) gastrointestinal surgery or troubles such as constipation, diarrhea or inflammatory bowel disease; (5) serious and unstable conditions such as a history of neurological disease or cardiopathy; (6) use of probiotics, antibiotics, immunomodulators or defecation drugs in the past month; (7) special diet such as a vegetarian diet; or (8) pregnancy.

### Clinical measurements

Demographic characteristics were obtained from a self-reported questionnaire, which involved age, sex, smoking status, and BMI. Drug history from self-report surveys included abstinence time and the age of initial MA use.

To collect clinical symptom data, several self-report scales were adopted for assessment. The Beck Depression Inventory (BDI) was to estimate the severity of depressive mood [43]; it consists of 13 items, and each item is scored on a scale ranging from 0 to 3. The severity of depression was determined based on BDI scores as follows: 0–4 (minimal depression), 5–7 (mild depression), 8–15 (moderate depression), and 16 (severe depression) [4]. The Beck Anxiety Inventory (BAI) was used to measure the severity of anxiety [44]. It contains 21 questions, and each item is scored on a scale ranging from 0 to 3. The severity of anxiety was determined based on BAI scores as follows: 8–15 (mild anxiety), 16–25 (moderate anxiety), and 26–63 (severe anxiety) [45].

The Pittsburgh Sleep Quality Index (PSQI) was applied to measure sleep habits across one month time, which contains 18 self-rated questions divided into seven components: sleep quality (P1), sleep latency (P2), sleep duration (P3), habitual sleep efficiency (P4), sleep disturbance (P5), use of sleeping medication (P6), and daytime dysfunction (P7) [46]. The PSQI total score (TS) ranges from 0 to 21 and a total score > 5 was considered to indicate “bad sleeper” [46]. According to previous research, a PSQI total score = 7 was also considered as the cut-off point [47, 48]. Therefore, the 70 enrolled MA users were divided into two groups: those with bad sleep quality (MA-BS; PSQI total score ≥ 7, *n* = 21) and those with good sleep quality (MA-GS; PSQI total score < 7, *n* = 49). The HCs were referred to as the HC-GS group. Visual Analog Scale (VAS) was utilized to estimate MA craving [49]; this scale is a 10-centimeter line ranging from 0 to 10 (0 representing “no craving” and 10 representing “highest craving”) [50].

### Fecal sample collection and DNA extraction

Fecal Samples were collected from the First Compulsory Drug Rehabilitation Center of Shenyang within 3 days after finishing the questionnaires and subsequently were kept in a -80℃ deep freeze freezer before DNA extraction. The fresh stool samples were stored in a fecal preservation solution (CW2654, CwBiotech, Beijing, China). The DNA was extracted by following the procedure instruction of the DNA extraction kit (MN® NucleoSpin 96 Soi kit, Germany).

### 16S rRNA sequencing

The 16S rRNA gene of gut bacteria was amplified in the V3-V4 regions by 2 round polymerase chain reaction (PCR) using specific primers (338F: 5’-ACTCCTACGGGAGGCAGCA-3’ and 806R: 5’-GGACTACHVGGGTWTCTAAT-3). The first round of PCR had the following parameters: 95 ºC for 5 min, followed by 25 cycles at 95 ºC for 30s, 50 ºC for 30s, 72 ºC for 40s, and then 72 ºC for 7 min for final extension while the second round PCR was under 98 ºC for 30s, followed by 10 cycles at 98 ºC for 10s, 65 ºC for 30s, 72 ºC for 40s, and a final extension at 70 ºC for 5 min. Finally, the amplicons were extracted from 1.8% agarose gels using a Monarch DNA extraction kit.

PCR products are purified by gel electrophoresis, qualified, and sequenced by the Illumina HiSeq 2500. The data acquired from Illumina HiSeq 2500 were first jointed and low-quality filtered using Fastp [51]. Cutadapt (Version 2.7.8) software was used to identify and remove primer sequences, and high-quality reads without primer sequences were obtained. Through Trimmomatic (Version 0.33) software [52], the raw reads obtained by sequencing were filtered to finally obtain high-quality reads. Then use USEAERCH (Version 10.0.240) [53] and VSEARCH (Version 2.15.2) [54] were used to generate abundance tables and species annotation tables of amplicon sequence variants (ASVs) and align sequences against the SILVA database (silva_16S_v123.fa) [55].

### Bioinformatic analysis

Alpha-diversity indices were calculated in R (Version 4.2.1), including the Chao1, ACE, Shannon index, and Simpson index. Then, alpha-diversity indices were compared between the MA-BS and MA-GS groups and the MA-GS and HC-GS groups. The Mann–Whitney U test was used to compare alpha-diversity indices, and the statistical significance was set at *p* < 0.05.

Beta diversity was computed in R (Version 4.2.1) and estimated by weighed Bray‒Curtis distance matrices. Differences in beta diversity were identified with permutational multivariate analysis of variance (PERMANOVA) with 999 permutations with the vegan R package (Version 2.6-4) and visualized with principal coordinate analysis (PCoA) with the ggplot2 R package (version 3.4.1). In the PERMANOVA, *p* < 0.05 was set as the significance threshold.

Linear discriminant analysis effect size (LEfSe) was also applied to identify prominently enriched taxa. Linear discriminant analysis (LDA) was used to identify taxa with significant differences. Taxa with LDA scores > 2 and *p* < 0.05 were considered significantly different. The analytical methods were performed on the online website Galaxy (https://huttenhower.sph.harvard.edu/galaxy/).

The Kyoto Encyclopedia of Genes and Genomes (KEGG) and Phylogenetic Investigation of Communities by Reconstruction of Unobserved States (PICRUSt, Version 2.4.1) [56] were used to predict the function of microbiota community. The differences in metabolic pathways were analyzed by STAMP (Version 2.1.3) [57] and identified by the Kruskal-Wallis rank-sum test. All comparisons were corrected for multiple testing (Benjamini–Hochberg correction, *q* < 0.05).

### Statistical analysis

R (version 4.2.1) and SPSS (version 26.0) were used to perform statistical analysis. Demographic and clinical characteristics were analyzed by SPSS. According to the normality of data distribution, data type, and the purpose of analysis, two-sample t tests, chi-square tests, or the Mann–Whitney U test were used to analyze age, sex, BMI, smoking status, initial age of MA use, duration of abstinence, clinical symptoms, relative abundances, and alpha diversity. Spearman partial correlation analysis was performed in R and used to determine the relationships among different PSQI component scores, other clinical symptoms (BDI, BAI, and VAS scores), and the relative abundance of taxa. Sex, age, BMI, PSQI total scores, BDI scores, BAI scores, and VAS scores were included as covariates according to analysis requirements. False discovery rate (FDR) correction was applied to the p value. The statistical significance of the above tests was set at *p* < 0.05 (two-tailed).

## Results

### Demographic, addiction, and clinical characteristics of participants

There were no differences in age, sex, BMI, smoking status, initial age of MA use, duration of abstinence, BDI scores, BAI scores, or VAS scores between the MA-GS and MA-BS groups, although there were significant group differences in the PSQI score and seven PSQI component scores. In addition, a larger proportion of MA abusers were male and smoked compared to that of healthy controls. Age, sex, BMI, initial age of MA use, duration of abstinence, PSQI score, six PSQI component scores (except daytime dysfunction), and BAI scores were not significantly different between the MA-GS and HC-GS groups, but there were significant differences in smoking status, daytime dysfunction, BDI scores, and VAS scores. All characteristics are listed in Tables 1 and 2.

**Table 1:** Demographic, addiction and clinical characteristics between MA-GS and MA-BS group

	MA-GS (*n* = 49)	MA-BS (*n* = 21)	Test value (t/χ2/Z)	*p* value
Demographic characteristics				
Age (years)	38.98 ± 9.65	39.95 ± 11.31	-0.367	0.715^**†**^
Gender (male)	35 (71.43%)	14 (66.67%)	0.158	0.690^#^
BMI (kg/m^2^)	25.95 ± 3.43	25.79 ± 4.68	0.164	0.870^**†**^
Smoking (Yes)	49 (100%)	20 (95.24%)	0.915	0.339^#^
Addiction characteristics				
Initial age of methamphetamine use	30.37 ± 8.57	30.05 ± 10.8	0.729	0.469^**†**^
Duration of abstinence (months)	2.00 (5.00)	2.00 (6.00)	-0.026	0.979^*^
Clinical characteristics				
Pittsburgh Sleep Quality Index (PSQI)	3.00 (4.00)	12.00 (4.00)	-6.630	< 0.001^*^
Sleep quality	0.00 (1.00)	2.00 (2.00)	-6.137	< 0.001^*^
Sleep latency	1.00 (1.00)	2.00 (1.00)	-5.117	< 0.001^*^
Sleep duration	1.00 (2.00)	2.00 (2.00)	-3.802	< 0.001^*^
Habitual sleep efficiency	0.00 (1.00)	1.00 (3.00)	-2.914	0.004^*^
Sleep disturbance	0.00 (1.00)	2.00 (1.00)	-6.178	< 0.001^*^
Use of sleeping medication	0.00 (0.00)	0.00 (2.00)	-4.171	0.003^*^
Daytime dysfunction	0.00 (0.00)	2.00 (1.00)	-6.038	< 0.001^*^
Beck Depression Inventory (BDI)	0.00 (7.00)	4.00 (11.00)	-1.301	0.193^*^
Beck Anxiety Inventory (BAI)	0.00 (4.00)	3.00 (9.00)	-1.599	0.110^*^
Visual Analog Scale (VAS)	0.00 (0.00)	0.00 (0.00)	-0.192	0.848^*^

Note: Data are presented as means ± standard deviations, percentages (%), or median (interquartile range)

^#^*P*-value for chi-square test, ^**†**^*P*-value for two-sample t-test. ^*^P-value for the Mann–Whitney U test

MA-GS, MA users with good sleep quality; MA-BS, MA users with bad sleep quality

**Table 2:** Demographic and clinical characteristics between MA-GS and HC-GS group

	MA-GS (*n* = 49)	HC-GS (*n* = 38)	Test value (t/χ2/Z)	*p* value
Demographic characteristics				
Age (years)	38.98 ± 9.65	42.74 ± 9.38	-1.824	0.072 ^**†**^
Gender (male)	35 (71.42%)	23 (60.53%)	1.145	0.285^#^
BMI (kg/m^2^)	25.95 ± 3.43	24.91 ± 2.82	1.499	0.137 ^**†**^
Smoking (Yes)	43 (87.76%)	4 (10.52%)	51.392	< 0.001^#^
Clinical characteristics				
Pittsburgh Sleep Quality Index (PSQI)	3.00 (4.00)	3.50 (3.00)	-1.110	0.267^*^
Sleep quality	0.00 (1.00)	0.00 (1.00)	-1.340	0.180^*^
Sleep latency	1.00 (1.00)	1.00 (1.00)	-0.353	0.724^*^
Sleep duration	1.00 (2.00)	1.00 (1.00)	-0.110	0.912^*^
Habitual sleep efficiency	0.00 (1.00)	0.00 (0.00)	-1.027	0.304^*^
Sleep disturbance	0.00 (1.00)	0.50 (1.00)	-1.509	0.131^*^
Use of sleeping medication	0.00 (0.00)	0.00 (0.00)	-0.181	0.856^*^
Daytime dysfunction	0.00 (0.00)	0.00 (1.00)	-2.127	0.033^*^
Beck Depression Inventory (BDI)	0.00 (7.00)	0.00 (1.00)	-2.034	0.042^*^
Beck Anxiety Inventory (BAI)	0.00 (4.00)	0.00 (2.00)	-0.618	0.536^*^
Visual Analog Scale (VAS)	0.00 (0.00)	0.00 (0.00)	-2.595	0.009^*^

Note: Data are presented as means ± standard deviations, percentages (%), or median (interquartile range)

^#^*P*-value for chi-square test, ^**†**^*P*-value for two-sample t-test. ^*^P-value for the Mann–Whitney U test

MA-GS, MA users with good sleep quality; HC-GS, healthy controls with good sleep quality

### Alpha-diversity and beta-diversity of the gut microbiota

**Fig. 1:**
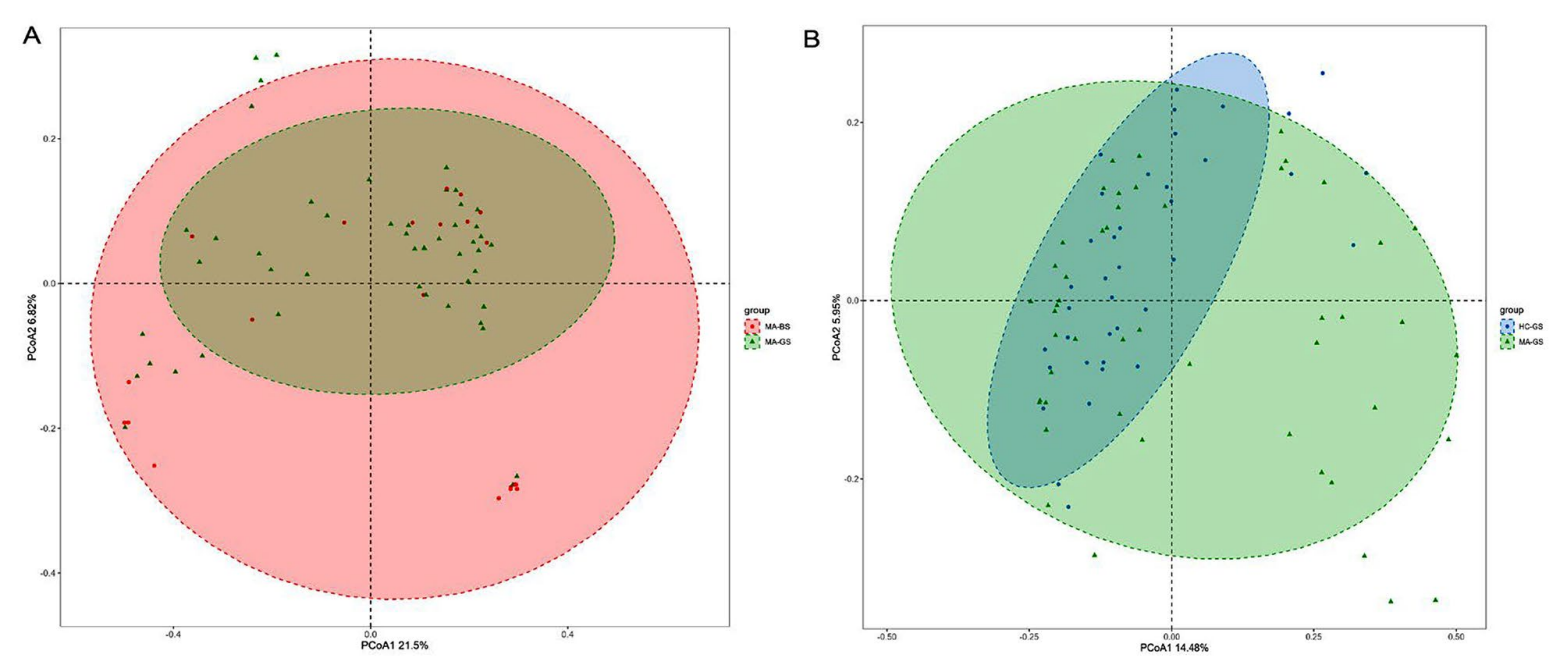
The beta diversity of the bacterial communities. Note: MA-GS, MA users with good sleep quality; MA-BS, MA users with bad sleep quality; HC-GS, healthy controls with good sleep quality

We used four indices to assess alpha diversity, including the Chao1, ACE, Shannon, and Simpson indices. Alpha diversity reflects the taxa richness and diversity of a single sample. The Chao1 and ACE indices measure taxa abundance, while the Shannon and Simpson’s indices evaluate abundance and community evenness. Regarding alpha diversity, there were no significant differences in the Chao1 (*Z* = -0.391, *p* = 0.696), ACE (*Z* = -0.391, *p* = 0.696), Shannon (*Z* = -0.365, *p* = 0.715), or Simpson indices (*Z* = -0.391, *p* = 0.696) between the MA-GS and MA-BS groups. The Chao1 (*Z* = -1.840, *p* = 0.066), ACE (*Z* = -1.866, *p* = 0.062), Shannon (*Z* = -1.429, *p* = 0.153), and Simpson indices (*Z* =-1.198, *p* = 0.231) were not significantly different between the MA-GS and HC-GS groups. Alpha diversity results are shown in Supplementary Figs. 1 and 2. Regarding beta diversity, we did not find significant differences between the MA-GS and MA-BS groups (R^2^ = 0.017, *p* = 0.225), while there were significant differences between the MA-GS and HC-GS groups (R^2^ = 0.024, *p* = 0.003). The PCoA visualization is shown in Fig. 1.

In order to control covariates such as age, gender, BMI, BDI scores, BAI scores, VAS scores, and smoking state, analysis of covariance (ANCOVA) was adopted to compare alpha diversity difference between MA-GS and MA-BS, MA-GS and HC-GS. There were no significant differences in the Chao1 (F = 0.125, *p* = 0.725), ACE (F = 0.160, *p* = 0.690), Shannon (F = 0.114, *p* = 0.737), or Simpson indices (F = 0.030, *p* = 0.863) between the MA-GS and MA-BS groups. The Chao1 (F = 0.350, *p* = 0.556), ACE (F = 0.295, *p* = 0.588), Shannon (F = 0.992, *p* = 0.340), and Simpson indices (F = 1.180, *p* = 0.281) were not significantly different between the MA-GS and HC-GS groups. Regarding beta diversity, we still control for the above covariates and did not find significant differences between the MA-GS and MA-BS groups (R^2^ = 0.017, *p* = 0.180), while there were significant differences between the MA-GS and HC-GS groups (R^2^ = 0.024, *p* = 0.003).

### Relative abundances of the gut microbiota

**Fig. 2:**
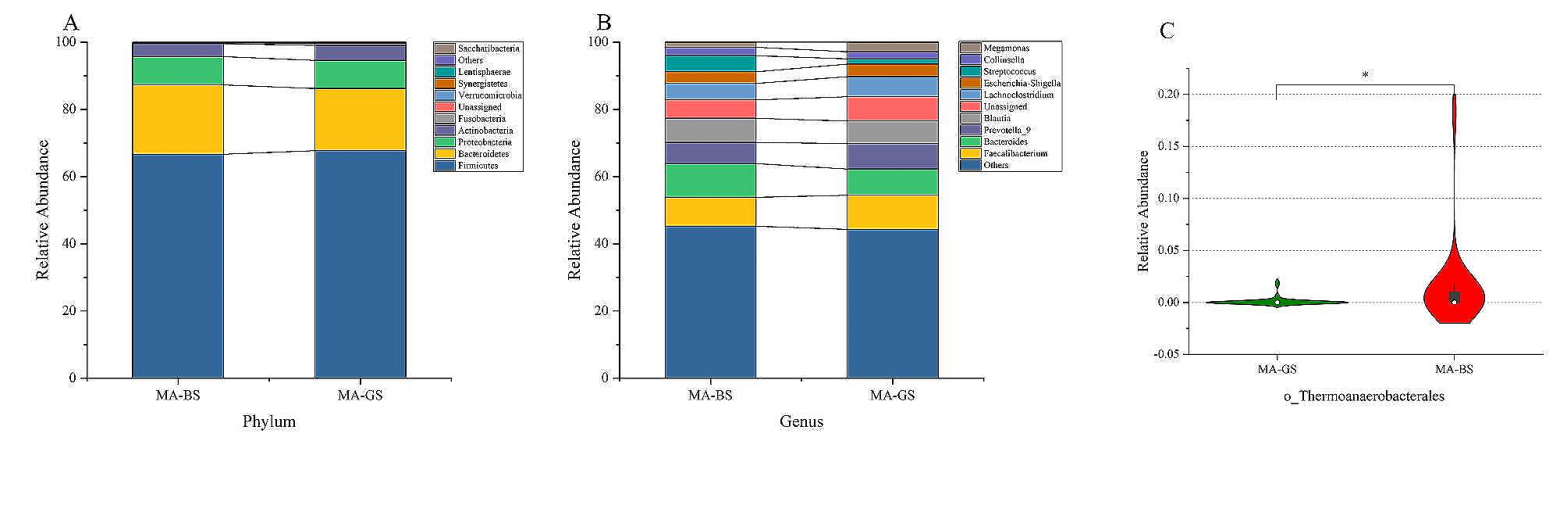
The top 10 of average gut microbiota relative abundance and the relative abundance about *Thermoanaerobacterales* between MA-GS and MA-BS group. Note: The top 10 of average gut microbiota relative abundance between MA-GS and MA-BS group at the phylum level **(A)** and the genus level **(B)**. **(C)** The relative abundance about *Thermoanaerobacterales* between MA-GS and MA-BS group at order level. MA-GS, MA users with good sleep quality; MA-BS, MA users with bad sleep quality. *p < 0.05, **p < 0.01, ***p < 0.001

Figure 2 show the average bacterial compositions of the MA-GS and MA-BS groups at the phylum and genus levels for the top 10 gut microbiota. The class, order, and family levels of microbial taxa in the MA-GS and MA-BS groups shown expressed in Supplementary Fig. 3, while the average bacterial compositions of the top 10 microbial taxa of the MA-GS and HC-GS groups are shown in Supplementary Fig. 4.

We observed differences in the relative abundances of several microorganisms. In the comparison of the MA-GS and MA-BS groups, *Thermoanaerobacterales* (*Z* = -3.229, *q* = 0.048) was discovered differ at the level order, but no differences at the other levels were observed. The MA-GS and HC-GS groups also significantly differed in the relative abundance results. At the phylum level, the results revealed different relative abundances of *Actinobacteria* (*Z* = -2.773, *q* = 0.02), *Bacteroidetes* (*Z* = -2.396, *q* = 0.0497), and *Firmicutes* (*Z* = -2.807, *q* = 0.02). At the class level, *Actinobacteria* (*Z* = -3.282, *q* = 0.012) was differed. At the order level, *Bifidobacteriales* (*Z* = -3.603, *q* = 0.004), *Micrococcales* (*Z* = -5.253, *q* < 0.001), and *Aeromonadales* (*Z* = -3.323, *q* = 0.009) differed between the two groups. At the family level, *Micrococcaceae* (*Z* = -5.023, *q* < 0.001) and *Bifidobacteriaceae* (*Z* = -3.603, *q* = 0.008) exhibited different relative abundances between the two groups. At the genus level, there were differences in the relative abundance of several microbiotas, including *Weissella* (Z = -3.835, q = 0.012), *Bifidobacterium* (*Z* = -3.612, *q* = 0.02), and *Faecalitalea* (*Z* = -3.453, *q* = 0.022). FDR correction was applied to all the above results. Further details about the differences in the relative abundances of taxa are shown in Fig. 2 and Supplementary Fig. 5. We used ANCOVA to respectively compare MA-GS and MA-BS, MA-GS and HC-GS, controlling for age, gender, BMI, BDI scores, BAI scores, VAS scores, and smoking state. After FDR correction, no gut microbiota exists statistical difference between MA-BS and MA-GS groups. Before FDR correction, there were statistical differences in order *Thermoanaerobacterales* (F = 4.638, *p* = 0.035) between MA-BS and MA-GS groups. Differences were found phylum *Firmicutes* (F = 11.666, *q* = 0.012) and genus *Incertae_Sedis* (F = 13.755, *q* = 0.042) between MA-GS and HC-GS groups, and FDR correction was performed.

**Fig. 3:**
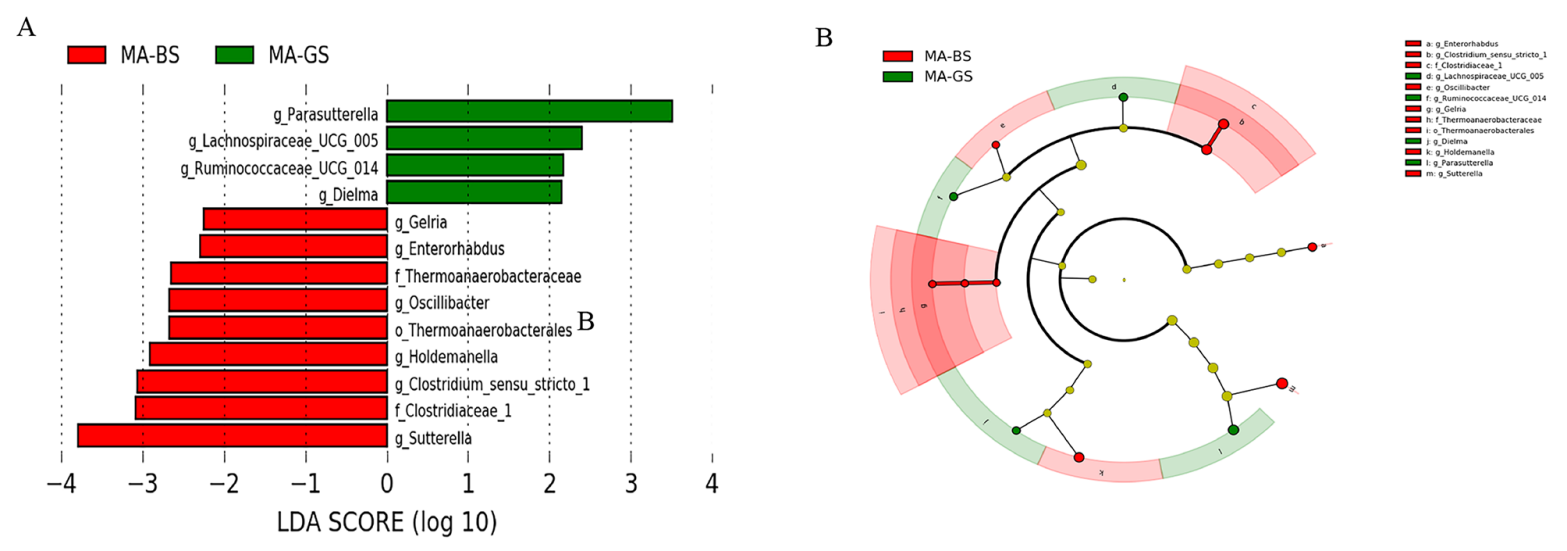
The taxa significant differences and the cladogram between MA-GS and MA-BS group. Note: **(A)** The taxa significant differences (LDA score > 2.0 and *p* < 0.05) between MA-GS and MA-BS group were detected by the LEfSe analysis. **(B)** The cladogram shows the differential taxa between the MA-GS and MA-BS group found in the LEfSe analysis. MA-GS, MA users with good sleep quality; MA-BS, MA users with bad sleep quality. P, phylum; c, class; o, order; f, family; g, genus

To further analyze the microbiota community structure, LEfSe analysis was applied. According to the LDA scores, the effect size of each microbiota is different between the MA-GS and MA-BS group, and the MA-GS and HC-GS group. The results of comparison between the MA-GS and MA-BS group found several microbiotas are associated with MA-BS at the order level (*Thermoanaerobacterales*), family level (*Thermoanaerobacteraceae* and *Clostridiaceae_1*), and genus level (*Clostridium_sensu_stricto_1, Enterorhabdus, Gelria, Holdemanella, Oscillibacter*, and *Sutterella*), which were discovered a significant increase in MA-BS group. As for the MA-GS group, *Dielma, Lachnospiraceae_UCG_005, Parasutterella*, and *Ruminococcaceae_UCG_014* at genus level were revealed enrichment. More details were exhibited in Fig. 3.

Regarding the LEfSe analysis comparative results between MA-GS and HC-GS, we selected the microbiotas with LDA > 4 shown as follows. The MA-GS group was significantly enriched at the phylum level (*Firmicutes*), class level (*Bacilli*), and genus level (*Lachnoclostridium* and *Escherichia_Shigella)*. Inversely, the HC-GS group was associated with the phylum *Bacteroidetes* and *Actinobacteria*, the class *Bacteroidia*, the order *Bacteroidales* and *Bifidobacteriales*, the family *Bacteroidaceae* and *Bifidobacteriaceae*, the genus *Bacteroides* and *Bifidobacterium.* More results were exhibited in Supplementary Fig. 6.

### Function prediction of the microbiota community by KEGG

To explore metabolic pathways related to MA users with bad sleep quality, PICRUSt and STAMP were used to map microbial genes to metabolic databases to infer microbial functions. No metabolic pathway was discovered to exhibit significant differences between the MA-GS and MA-BS groups after FDR correction. Regarding the microbial functions of MA users with good sleep quality, there exists metabolism difference with the HC-GS group after FDR correction, which includes biosynthesis of other secondary metabolites and amino acid metabolism. These results are shown in Supplementary Fig. 7.

### Sleep quality was related to the gut microbiota

**Fig. 4:**
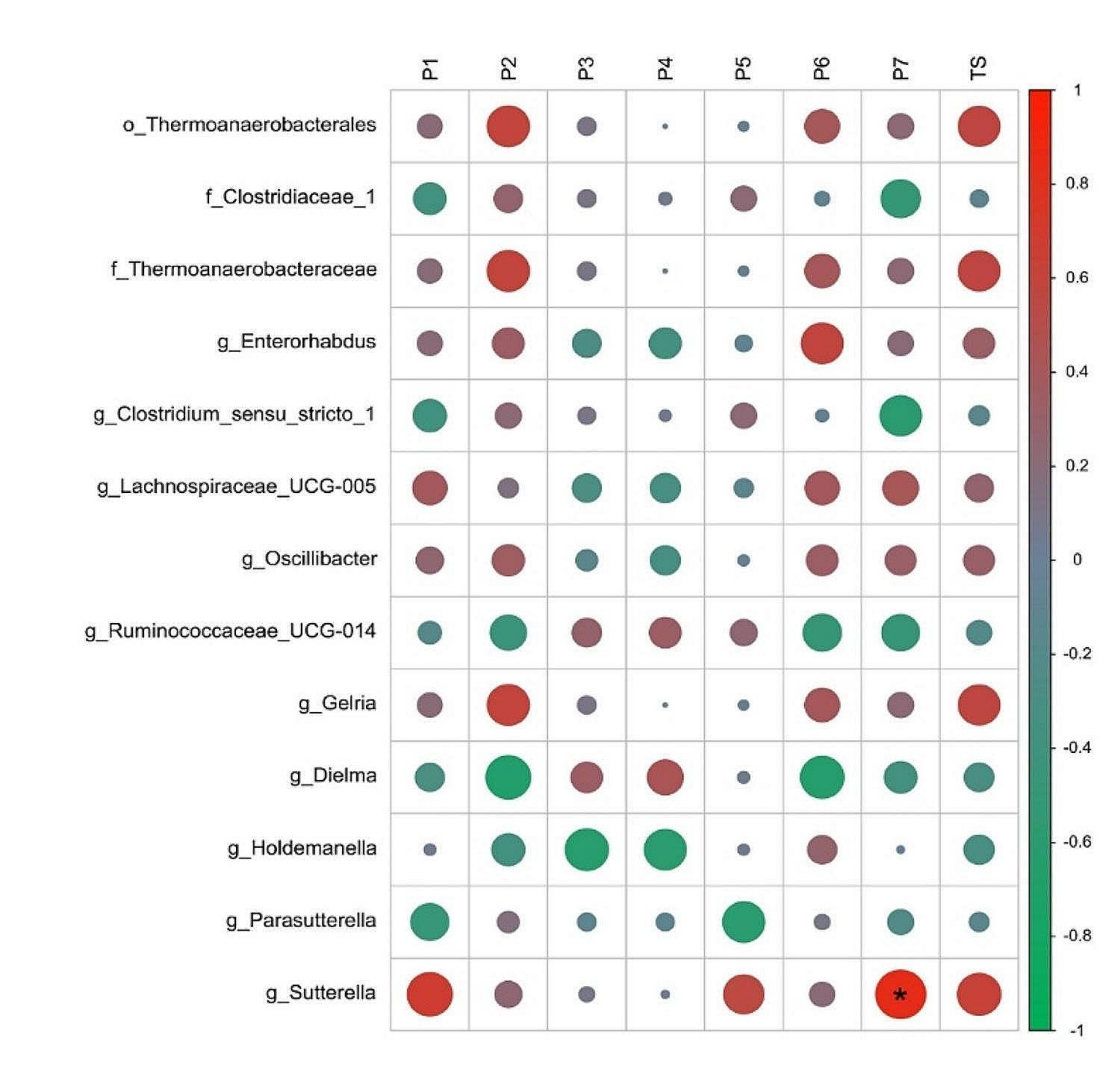
The partial spearman correlation between relative abundance of signature the gut microbiota and the Pittsburgh Sleep Quality Index (PSQI) scores in MA-BS group. Note: P1, sleep quality; P2, sleep latency; P3, sleep duration; P4, habitual sleep efficiency; P5, sleep disturbance; P6, use of sleeping medication; P7, and daytime dysfunction; TS, total scores. MA-BS, MA users with bad sleep quality. P, phylum; c, class; o, order; f, family; g, genus. *p < 0.05, **p < 0.01, ***p < 0.001

To analyze the relationship between sleep quality and the gut microbiota in the MA-BS group, we selected the bacterial taxa with LDA scores > 2 identified by the comparison between the MA-GS and MA-BS groups. Therefore, 13 gut microbes were considered for analysis. Based on the seven components of the PSQI and the PSQI total score, the scores for eight components were calculated: sleep quality (P1), sleep latency (P2), sleep duration (P3), habitual sleep efficiency (P4), sleep disturbance (P5), use of sleeping medication (P6), daytime dysfunction (P7), and total scores (TS). Spearman partial correlation analyses were conducted with sex, age, BMI, BDI scores, BAI scores, and VAS scores as covariates. Finally, the results showed that the relative abundance of *Sutterella*, belonging to the family *Alcaligenaceae*, order *Burkholderiales*, class *Betaproteobacteria*, and phylum *Proteobacteria*, was significantly positively correlated with P7 scores (*r* = 0.83, *p* = 0.011). FDR correction was applied to all the above results. The heatmap is shown in Fig. 4.

In addition, the bacterial taxa with LDA scores > 4 identified by the comparison between the MA-GS and HC-GS groups were adopted to analyze the correlation between microbial taxa relative abundance and clinical symptoms in the MA-GS group. After controlling for the covariates sex, age, BMI, BDI scores, BAI scores, and TS, the results showed that the VAS score was negatively correlated with the relative abundance of *Bacteroides* (*r* = -0.349, *p* = 0.022), which belonged to the family *Bacteroidaceae*, order *Bacteroidales*, class *Bacteroidia*, and phylum *Bacteroidetes*. Other correlations were not discovered, and FDR correction was not applied to the above results.

## Discussion

As far as we know, the present research is the first time to investigate the gut microbiota features of MA users with varying degrees of sleep quality by using the 16S rRNA Sequencing method. In addition, the gut microbiota features of MA users without sleep problems were explored by comparing them with healthy controls. By comparing the MA-GS and MA-BS groups, the present research found that there did not exist differences in alpha-diversity and beta-diversity of gut microbiota, but the relative abundances of gut microbiota were altered. Meanwhiles, the *Sutterella* genus was discovered positively correlated with daytime dysfunction. We also compared the distinctions between MA users and HCs, excluding the effect of sleep problems. The results elucidated that alpha-diversity did not exist statistical difference while discrepancies in beta-diversity were the opposite. In addition, relative abundances of different taxa were discovered to be changed. These findings above suggested that gut microbiota may play an important role in MA users and their sleep quality.

In the present study, no significant difference was observed in terms of alpha diversity between MA-GS and MA-BS groups or between MA-GS and HC-GS groups, which is consistent with previous studies [34, 58]. Our findings suggest a notable dissimilarity between the beta diversity of the MA-GS and HC-GS groups, which is consistent with the literature [33]. This suggested that there may exist a large difference in the composition of gut microbiota between MA users and healthy people.

According to the current results, the relative abundance of *Parasutterella* was decreased in MA users with bad sleep quality, and a previous study demonstrated that *Parasutterella* was reduced in humans after sleep deprivation [59]. We also found *Ruminococcaceae_UCG_014* and *Lachnospiraceae_UCG_005* enriched in MA users with good sleep quality. *Ruminococcaceae* was considered to decrease in insomnia patients [60] and be positively associated with sleep quality [41]. Meanwhile *Ruminococcaceae* and *Lachnospiraceae* can produce short-chain fatty acid (SCFA), which has anti-inflammatory effects and are beneficial to human health [61, 62]. Family *Thermoanaerobacteraceae* and genus *Sutterella* were discovered increasement in MA with bad sleep quality. Additionally, *Sutterella* was verified to be positively associated with daytime dysfunction in MA users in the current results. However, other findings about sleep problems are inconsistent with our results. We speculate that this may have been caused by MA consumption and *Sutterella* may be a special gut microbiota in MA users with daytime dysfunction. Most of the current reports related to *Sutterella* have focused on autism and some intestinal disorders, and it is not yet clear what the consequences of an increase in *Sutterella* relative abundance are, but it is possible that under specific conditions these bacteria could cause infection [63–67]. Furthermore, there existed several experiments to improve sleep quality by adding probiotics, which have acquired satisfactory effectiveness [68, 69]. In summary, our study, and former research all demonstrated the significance of gut flora in sleep quality, helping us to better improve the sleep problems of MA abusers.

Based on the results of the comparison between the MA-GS and HC-GS groups, which has eliminated the interference of sleep problems, we found *Bifidobacterium* and *Bacteroides* at the genus level reduced while family *Micrococcaceae, order Aeromonadales*, genus *Faecalitalea*, and genus *Escherichia_Shigella* increased in MA users. *Bifidobacterium* and *Bacteroides* were considered as a beneficial role in human health, which have been associated with metabolites such as short-chain fatty acids, and bacteriocins that potentially promoted health state or neurodevelopment [70–72]. *Aeromonadales* may cause gastroenteritis and extraintestinal diseases [73], and *Faecalitalea* was used to identify autism spectrum disorder (ASD) and HC [74], and *Escherichia_Shigella* was associated with human diseases such as Tuberculous meningitis [75] or cognitively impaired [76]. These gut microorganisms were considered to be harmful to health. Therefore, we discovered beneficial bacteria lessened while pernicious bacteria increased. Nevertheless, current studies on gut microbiota alterations in methamphetamine users are highly heterogeneous, and our results are inconsistent with those of previous studies [33, 34, 58]. We inferred that the reason why the results are distinct may be that our study excluded sleep problems, homosexual behaviors, and the addition of women using MA. Anyway, both our results and previous literature confirmed that the gut microbiota is altered in MA users.

Our study also has several limitations. First, the PSQI was employed to evaluate sleep quality, which is a self-reported inventory. Future studies should apply more objective measurements, such as polysomnography, to improve the precision of sleep quality assessments. Second, we only compared groups in terms of the gut microbiota tested by 16S rRNA sequencing, but such differences do not explain the underlying mechanism, which suggests that testing to identify inflammatory factors or products of metabolism, and adopt shotgun sequencing to detect gut microbiota may be needed to further explain the role of the gut microbiota in MA users with bad sleep quality. Third, demographic characteristics and drug history were obtained from self-reported questionnaires, which might exist an information bias and suggest that more objective information is needed for future research. In addition, we did not obtain information about daily diet and exercise, and we will include this information in future research. Lastly, our study was a cross-sectional study with a small sample size in each group. The sample size should be expanded in the future, and relevant longitudinal work should be conducted.

In conclusion, the present study investigated gut microbiota alterations in MA users. Moreover, we further revealed that the genus *Sutterella* may be related to daytime dysfunction in MA users, suggesting that the genus *Sutterella* may be a biomarker for bad sleep quality in MA users.

### Electronic supplementary material

Below is the link to the electronic supplementary material.

Supplementary Material 1

## Data Availability

The data presented in the study are deposited in the National Center for Biotechnology Information (NCBI) BioProject database with project number PRJNA970410.
