# Supplementary material for: Alterations in the fecal microbiota of methamphetamine users with bad sleep quality during abstinence
Source: BMC Psychiatry. 2024 Apr 25;24:324. doi: 10.1186/s12888-024-05773-5 (PMC11046801; doi:10.1186/s12888-024-05773-5)
Supplement: Supplementary file 1 — Supplementary Material 1 [file 12888_2024_5773_MOESM1_ESM.docx]

**Alterations in the Fecal Microbiota of Methamphetamine Users with Bad Sleep Quality during Abstinence**

Figure 1| The alpha diversity between MA-GS and MA-BS. MA-GS, MA users with good sleep quality; MA-BS, MA users with bad sleep quality. NS, no statistical significance.

Figure 2| The alpha diversity between MA-GS and HC-GS. MA-GS, MA users with good sleep quality; HC-GS, healthy controls with good sleep quality. NS, no statistical significance.

Figure 3| The top 10 in average gut microbiota relative abundance between MA-GS and MA-BS group at the class level (A), the order level (B), and the family level (C). MA-GS, MA users with good sleep quality; MA-BS, MA users with bad sleep quality.

Figure 4| The top 10 in average gut microbiota relative abundance between MA-GS and HC-GS group at the phylum level (A), the class level (B), the order level (C), the family level (D) and the genus level (E). MA-GS, MA users with good sleep quality; HC-GS, healthy controls with good sleep quality.

Figure 5| The relative abundance about different bacterial taxa between MA-GS and HC-GS group. MA-GS, MA users with good sleep quality; HC-GS, healthy controls with good sleep quality. P, phylum; o, order; f, family; g, genus. *p < 0.05, **p < 0.01, ***p < 0.001.

Figure 6| (A) The taxa significant differences (LDA score>2.0 and p<0.05) between MA-GS and HC-GS group were detected by the LEfSe analysis. (B) The cladogram shows the differential taxa between the MA-GS and HC-GS group found in the

LEfSe analysis. MA-GS, MA users with good sleep quality; HC-GS, healthy controls with good sleep quality. P, phylum; c, class; o, order; f, family; g, genus.

Figure 7| Functional prediction analysis of the gut microbiota between MA-GS and HC-GS group. The bar graph represents the pathways with a significant difference in relative abundance between the two groups. The figure on the right shows 95.0% confidence intervals and q-value after FDR correction. MA-GS, MA users with good sleep quality; HC-GS, healthy controls with good sleep quality.
